# Autophagy manipulation as a strategy for efficient anticancer therapies: possible consequences

**DOI:** 10.1186/s13046-019-1275-z

**Published:** 2019-06-14

**Authors:** Mara Cirone, Maria Saveria Gilardini Montani, Marisa Granato, Alessia Garufi, Alberto Faggioni, Gabriella D’Orazi

**Affiliations:** 1 Department of Experimental Medicine, “Sapienza” University of Rome, Rome, Italy; 2 Laboratory affiliated to Istituto Pasteur Italia-Fondazione Cenci Bolognetti, Rome, Italy; 3 Department of Medical Science, University ‘G. D’Annunzio’, 66013 Chieti, Italy; 4 Department of Research, IRCCS Regina Elena National Cancer Institute, 00144 Rome, Italy

**Keywords:** Autophagy, Cancer, Immunogenic cell death (ICD), Endoplasmic reticulum (ER stress), Unfolded protein response (UPR), Chloroquine (CQ), Hydroxichloroquine (HCQ), p53, HSF1, NRF2

## Abstract

Autophagy is a catabolic process whose activation may help cancer cells to adapt to cellular stress although, in some instances, it can induce cell death. Autophagy stimulation or inhibition has been considered an opportunity to treat cancer, especially in combination with anticancer therapies, although autophagy manipulation may be viewed as controversial. Thus, whether to induce or to inhibit autophagy may be the best option in the different cancer patients is still matter of debate. Her we will recapitulate the possible advantages or disadvantages of manipulating autophagy in cancer, not only with the aim to obtain cancer cell death and disable oncogenes, but also to evaluate its interplay with the immune response which is fundamental for the success of anticancer therapies.

## Background

Macroautophagy, hereafter referred as autophagy, is a bulk degradative process up-regulated under stressful conditions, playing a central role in cellular homeostasis [1]. Autophagy usually helps cancer cells to cope with the shortage of nutrients and with the hypoxic conditions in which they are forced to survive. The modulation of autophagy may play dual roles in tumor suppression and promotion [2, 3]. Its induction is generally considered a valid option in cancer prevention [4], particularly because through a selective form of autophagy, that is the mitophagy, cells ride out of damaged mitochondria, the main producers of reactive oxygen species (ROS) that cause DNA mutations [5]. Autophagy modulators have been used as new anticancer strategy [3, 6], although how to manipulate autophagy to improve the treatment of established cancers is still not clear. Recently, a role of autophagy in the regulation the function of the cells present in the tumor microenvironment such as cancer-associated fibroblasts and immune cells has been highlighted, making the issue of autophagy manipulation even more challenging [7, 8]. Even if many reviews have been published in the last years about autophagy and cancer, here, we will try to recapitulate the multifaceted role of autophagy in cancer therapy and how its manipulation may impact immune response that plays an essential role in tumor regression.

### Interplay between autophagy and immune system in anticancer therapies

**Fig. 1:**
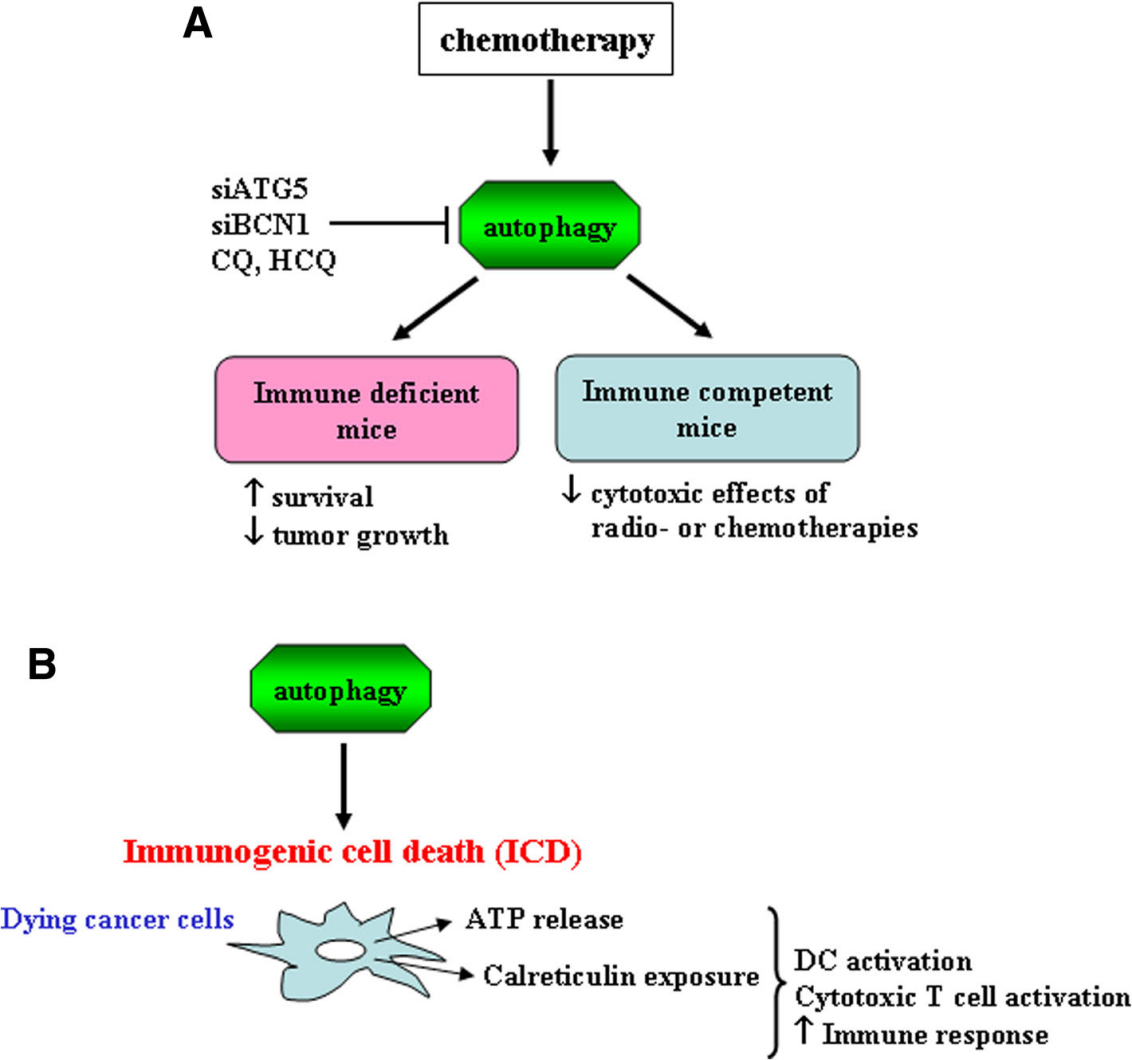
**a** Schematic representation of blockade of chemotherapies-induced autophagy and the relative outcome in tumor xenografts of immune deficient mice or immune competent mice models. **b** Schematic representation of immunogenic cell death (ICD) induced by autophagy. Dying cancer cells because chemotherapies activate autophagy that allows ATP release and calreticulin exposure that favor the activation of the immune response

The inhibition of autophagy has been pursued as a possible avenue to treat cancer, considering that autophagy represents a mechanism of adaption to stress especially when exacerbated by chemotherapies [9]. Indeed, excluding the rare and debated cases in which chemotherapies may induce an autophagic cell death [10], autophagy is triggered along with apoptosis as a pro-survival mechanism, as also evidenced by our studies [11–16]. Based on this knowledge, in vivo studies have started to employ autophagy inhibitors, such as inhibitors of the lysosomal protease and anti-malaric drugs, Chloroquine (CQ) or Hydroxichloroquine (HCQ), to treat cancer, more often in combination with chemotherapies able to induce autophagy [17–19]. Such combinations, mainly used to treat cancer in xenograft mouse models, have registered some successes in controlling tumor growth and prolonging host survival [20–22]. However, in order to avoid tumor rejection, immune deficient mice have been used for these experiments, thus cutting out the possibility to explore the direct and indirect role of autophagy inhibitors on the cells of the immune system [8]. Moving forward, the impact of autophagy inhibition in combination with chemotherapy has been explored also in immune competent mice. Surprisingly, these studies demonstrate that the depletion of essential autophagy-relevant gene products such as autophagy related (ATG) 5 or beclin 1 (BECN1) [1–3], although increase the cancer cytotoxic effect of therapy in vitro and in vivo in immune deficient mice, reduce the efficacy of radiotherapy or chemotherapy in immune competent mice [23] (Fig. 1a). These findings were somehow surprising because it raised many questions about the likely key role of the immune response for efficient anticancer therapies in the course of autophagy manipulation. In the mean time, several molecules exposed on the cancer cell surface or released by dying cancer cells upon chemotherapies, were discovered to elicit an immunogenic dell death (ICD) able to activate the immune system [24, 25]. In this regard, our studies identified Calreticulin and Heat Shock Protein (HSP) 90 as the Damage Associated Molecular Patterns (DAMPs) exposed on the surface of dying lymphoma cells treated by Bortezomib, and the CD91 as the receptor molecule involved in their recognition by dendritic cells (DCs) [26, 27]. DCs are powerful antigen-presenting cells (APCs) that play a pivotal role initiating a specific immune response and in the eradication of apoptotic cancer cells by mediating the cross-presentation of tumor antigens to the cytotoxic T cells, therefore, their function is fundamental for immune response activation [28]. Further investigations have highlighted that autophagy strongly contributes to the immunogenicity of cell death, promoting the release of adenosine triphosphate (ATP), a DAMP that plays a key role in immune cell activation [23, 29, 30] (Fig. 1b). These findings could explain why the combination of chemotherapy with autophagy inhibitors did not give the expected result in tumor models in immune competent mice, as it now clear enough that the contribution of the immune response is essential for a successful antitumor therapy.

Despite the unclear role of autophagy inhibition in improving the outcome of chemotherapies, clinical trials have started to use CQ or HCQ, mainly in combination with chemotherapies, to treat cancer patients [9, 31, 32]. The results so far obtained have been quite disappointing and the treatment failure may be explained also by the reduction of autophagy-induced ATP release, and by the fact that these anti-malaric drugs inhibit lysosomal acidification, thus may affect many other important cellular processes other than autophagy [33]. Moreover, when systemically administrated, CQ or HCQ may have several side effects [34] and act on immune cells suppressing their functions, i.e. stimulating the T regulatory cells (Treg) [35], altering class II antigen presentation or cross-presentation by DCs [36] or even impairing DC formation, all mechanisms inducing suppression of the immune response [37]. Interestingly, the reduction of autophagy in monocytes represents a strategy through which the human oncogenic gammaherpesviruses Epstein-Barr virus (EBV) and Kaposi’s sarcoma-associated herpesvirus (KSHV) alter monocyte differentiation into DCs, to escape from immune recognition, as also demonstrated by our studies [38–40]. In line with the evidences indicating that autophagy is required for an effective immune response and for the activation of immune system in the course of anticancer chemotherapies, we have found that autophagy inhibitor CQ abrogates the cytotoxic effect of curcumin against breast cancer in immune competent mice while increases it in immune deficient mice [40]. These findings point out, once again, that autophagy inhibition reduces the success of anticancer therapy in the presence of a functional immune system. Moreover, this study evidenced that CQ counteracts the curcumin down-regulation of Hypoxia Inducing Factor (HIF)-1, the main effector of cellular response to hypoxia involved in cancer progression and chemoresistance [41], and that sustained HIF-1 activation correlates with higher infiltrate of immune suppressive Treg cells in the tumor bed of curcumin plus CQ-treated mice [40]. In agreement, previous studies have shown that HIF-1 could be degraded through the lysosomal route [42, 43], suggesting that autophagy inhibition by CQ may interfere with HIF-1 degradation promoted by curcumin and sustain its oncogenic function for tumor progression.

### Autophagy and oncogenes degradation

**Fig. 2:**
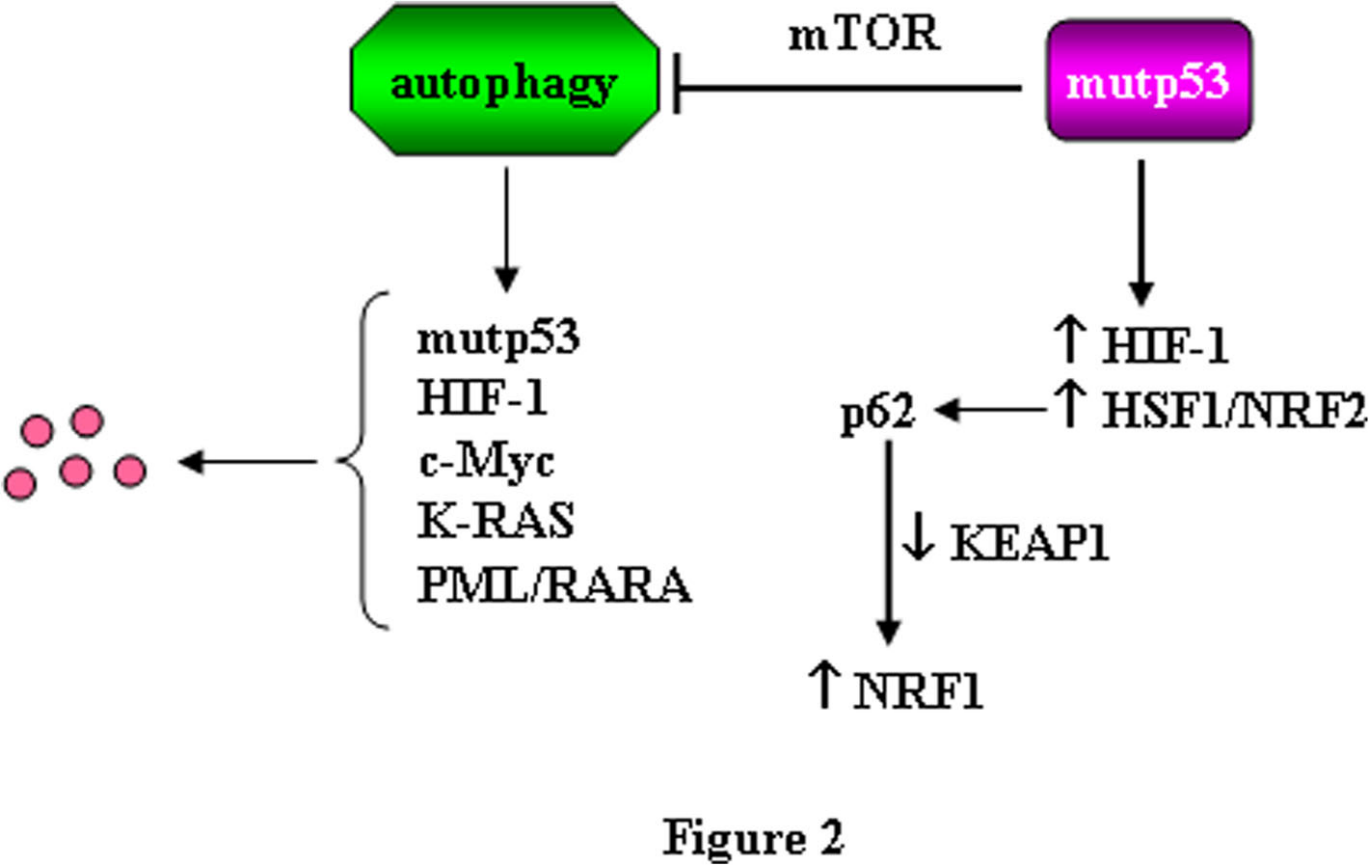
Schematic representation of the effect of autophagy on oncogenes degradation. The role of mutp53 in blocking autophagy and sustaining oncogenes activation is also shown

Here we come to another important and probably under-estimated role of autophagy in cancer, namely its capacity to degrade molecules involved in tumor survival, progression or chemoresistance, such as oncogenes or mutated oncosuppressor genes. At this purpose, our and other’s laboratories have shown that some mutant (mut) p53 proteins, that acquire pro-oncogenic functions (gain-of-function, GOF) [44], may undergo degradation through autophagy [45–48] or through chaperone-mediated autophagy (CMA) [49], both inhibited by the use of CQ and HCQ. While wild-type p53 has been reported to induce autophagy, mutp53 has been reported to reduce autophagy, especially when it is localized in the cytoplasm as a self-protective mechanism [16, 50], or through stimulation of the mammalian target of rapamycin (mTOR) pathway, sustaining tumor progression [51, 52]. Interestingly, mutp53 may activate HIF-1 [53] and it could be speculated that the inhibition of autophagy by mutp53 might promote HIF-1 activation, given that HIF-1 is degraded through the lysosomal route [42, 43]. The best described mechanism of mutp53 GOF is its ability to interact with transcription factors, remodelling the cancer cell transcriptome and proteome in such a way to support cancer cell survival, tumor progression, invasion, metastasis and chemoresistance [54]. Thus, other than interacting with HIF-1, mutp53 may interact and contribute to the activation of Heat Shock Factor 1 (HSF1) [55], a transcription factor that maintains cellular homeostasis by stress-mediated induction of HSP and coordinates cellular processes critical for malignancy such as metastasis and inhibition of apoptosis [56, 57]. Interestingly HSF1, activated in response to proteotoxic stress and basally activated in cancer cells [55], has been shown to be degraded through autophagy [58]. HSF1 can engage a cross-talk with nuclear factor erytroid 2 like (NRF2/NFE2L2) [59], the main transcription factor regulating the antioxidant response [60]. HSF1 and NRF2 regulate autophagy [60] and both promote the transcription of sequestosome 1/p62 (SQSTM1/p62) [59], a protein that is indeed up-regulated in stressful conditions. SQSTM1/p62 is mainly degraded through autophagy and thus is considered a marker to evaluate the completeness of the autophagic flux, as it accumulates when autophagy is inhibited [1]. SQSTM1/p62 may control a variety of other cellular processes involved in cell death or survival decision [61, 62]. Importantly, SQSTM1/p62 may stabilize NRF2, by degradation of NRF2 negative regulator kelch like ECH associated protein (Keap)1, thus linking autophagy to the anti-oxidant response [63] (Fig. 2). NRF2 is another transcription factor with which mutp53 may interact, promoting the transcription of pro-survival antioxidant enzymes [54] and this interplay with oncogenes further sustain tumor progression [64]. Included in the list of oncogenic transcription factors interconnected with mutp53 [65] and regulated by autophagy there is also c-myc, thus our studies showed that autophagy contributes to its degradation in Burkitt’s lymphoma cells treated with quercetin [66]. Furthermore, other oncogenic proteins such as K-RAS [67] and PML/RARA [68] can be degraded through autophagy and interact with mutp53 [69, 70] (Fig. 2). It is somehow intriguing that the expression of mutp53 and of many other oncogenic proteins interconnected with it may be regulated by autophagy and/or may regulate autophagy. The number of these oncogenic proteins is increasing, suggesting that other molecules involved in cancer development, survival and progression could come out to be regulated by autophagy. Considering that the oncogenic pathways may activate each other and that such cross-talk, besides cancer cells, may influence the function of immune cells, many other important effects of autophagy manipulation could be discovered. For example, it has been recently shown that PI3K/AKT/mTOR pathway, the master regulator of autophagy, often activated in cancer cells, may be involved in the up-regulation of the immune check-point inhibitor PD-L1 [71] whose expression on the tumor cells, by interacting with PD-1 on T cell surface, induces T cell exhaustion [72, 73]. It will be important to further explore the interplay between autophagy and PD-L1 expression, for example in cancer cells harboring mutp53, whose expression inhibits autophagy and activates mTOR.

### Interplay between autophagy, endoplasmic reticulum (ER) stress and unfolded protein response (UPR)

**Fig. 3:**
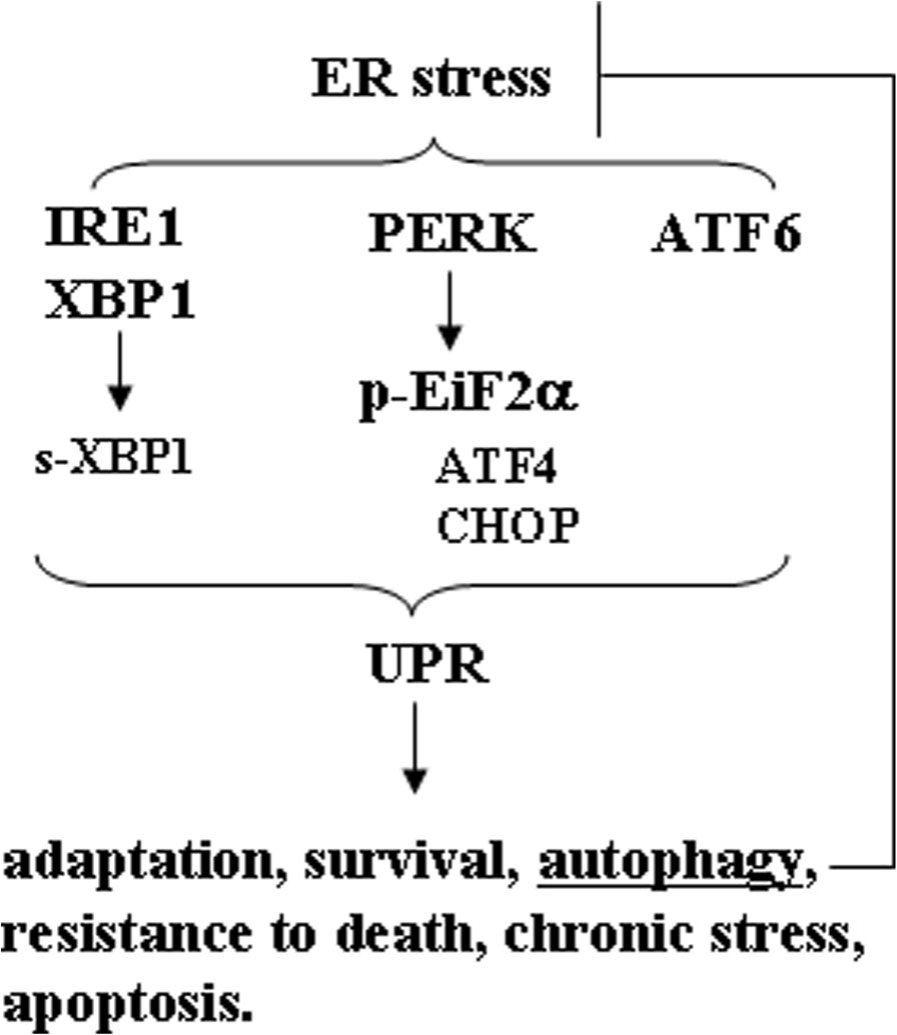
Molecular mechanisms of ER stress unfolded protein response (UPR) pathways. The three main sensors of UPR, localized at the ER membrane, and activated are inositol-requiring enzyme 1α (IRE1α), PKR-like ER kinase (PERK), and activating transcription factor 6 (ATF6). The autophagy induction alleviates the ER stress

Last but not least, it must be considered the interplay between autophagy, Endoplasmic Reticulum (ER) stress and Unfolded protein response (UPR) in the regulation of cancer cell survival [74]. Many reviews have been recently published elucidating the role of ER stress, UPR and autophagy in cancer [75–78]. The ER stress is induced by several cellular stresses that activates UPR to reduce the amount of misfolded proteins through ubiquitin-proteasome-dependent ERAD (ER-associated degeneration) and autophagy activation that restores ER homeostasis [75, 76, 78]. Under prolonged and irreversible ER stress, cells undergo apoptosis (Fig. 3) [75, 76, 78]. The UPR is indeed a transcriptional program that induces adaptation, survival, transformation, angiogenesis and resistance to cell death through three main sensors localized at the ER membrane: the inositol-requiring enzyme 1α (IRE1α), PKR-like ER kinase (PERK) and the activating transcription factor 6 (ATF6) [79]. IRE1α trans-autophosphorylation induces cleavage of XB1 leading to expression of the transcription factor XBP1s that regulates the expression of genes related with folding, entry of proteins to the ER, ER-associated degradation (ERAD) and biogenesis of ER and Golgi; PERK activation favours the phosphorylation of eIF2α (eukaryotic translation initiation factor 2α) and the selective translation of ATF4 (activating transcription factor 4), regulating the expression genes involved in folding, oxidative stress and amino acid metabolism; ATF6 translocates to the nucleus to induce the transcription of genes involved in ER homeostasis, and ERAD components (Fig. 3) [75–79]. ER stress is known to promote autophagy, and although the interplay between them remains still to be fully elucidated, the activation of UPR arms EIF2α and IRE1 have been reported to trigger autophagy [76, 80]. On the other hand, the inhibition of autophagy may exacerbate ER stress [80], altering the activation of UPR arms, leading for example to the up-regulation of the pro-apoptotic molecule C/EBP homologous protein (CHOP). Of note, CHOP can activate Cyclooxigenase (COX)-2 that in turn may promote the release of Prostaglandin (PG) E2, a DAMP that induces immune suppression [81, 82]. Moreover, ER stress in cancer cells promotes the release of factors such as ROS that may transfer ER stress from tumor cells to the immune cells, such as DCs, in the tumor environment. This event may activate the endoribonucleasic activity of IRE1α in DC, inducing the splicing of X-box binding protein (XBP1s). The formation of XBP1s may in turn promote an abnormal accumulation of peroxidized lipids, strongly impairing the immune function of DCs [83]. XBP1s’ activation and the up-regulation of CHOP have been also observed in myeloid suppressive DCs (MDSCs) present in the tumor environment [84]. Interestingly, it has been reported that ER stress can be transferred from cancer cells also to macrophages, promoting their polarization into M2 phenotype [85], tumor-associated macrophages that support instead of fighting tumor [86].

## Conclusions

Based on the findings reported by the majority of studies in this field, it seems that autophagy induction rather than autophagy inhibition could be exploited to improve the outcome of cancer treatment, at least in immune competent hosts. Therefore, *nutraceuticals*, exercise, calory restriction or calory restriction mimetics (such as metformin), all able to induce autophagy, are being considered as a possible alternative avenue to treat cancer in combination with chemotherapies [87, 88]. In addition, just to make this complicated field more complicated, it is emerging that inhibiting autophagy specifically in cancer cells may enhances the abscopal response to radiation therapy, that is, the ability of localized radiation to trigger systemic antitumor effects [89, 90]. thus suggesting that selective autophagy inhibition in cancer cells and systemic induction of autophagy could be combined to improve the outcome of anti-cancer therapy. Considering the role of autophagy in regulating the expression of oncogenes and modulating the function of the cells of the tumor environment such as fibroblasts and immune cells, more questions than answers have been raised by this review. Therefore, more investigations are needed to further clarify the possible consequences of autophagy manipulation in cancer therapy.

## Data Availability

All data analysed in this study are included in this published article.

## Abbreviations

APCs: Antigen-presenting cells
ATG5: Autophagy related 5
ATP: Adenosine triphosphate
BECN1: Beclin 1
CHOP: C/EBP homologous protein
COX-2: Cyclooxigenase-2
CQ: Chlororoquine
DAMPs: Damage Associated Molecular Patterns
DCs: dendritic cells
EBV: Epstein-Barr virus
EIF2α: Eukaryotic translation initiation factor 2α
ER: Endoplasmic Reticulum
HCQ: Hydroxichloroquine
HIF-1: Hypoxia Inducible Factor-1
HSF1: Heat Shock Factor 1
HSP90: Heat shock protein 90
ICD: Immunogenic dell death
Keap1: kelch like ECH associated protein 1
KSHV: Kaposi’s sarcoma-associated herpesvirus
MDSCs: myeloid suppressive DCs
mTOR: Mammalian target of rapamycin
NRF2: Nuclear factor erytroid 2 like
PGE2: Prostaglandin E2
ROS: Reactive oxygen species
SQSTM1: Sequestosome 1
UPR: Unfolded protein response
XBP1: X-box binding protein 1
